# Sperm Membrane: Molecular Implications and Strategies for Cryopreservation in Productive Species

**DOI:** 10.3390/ani15121808

**Published:** 2025-06-19

**Authors:** Macarena Castro, Karla Leal, Felipe Pezo, María José Contreras

**Affiliations:** 1 Doctorado en Ciencias Aplicadas, Facultad de Ingeniería, Universidad Autónoma de Chile, Temuco 4810101, Chile; 2 Instituto de Ciencias Aplicadas, Facultad de Ingeniería, Universidad Autónoma de Chile, Temuco 4810101, Chile; 3 Facultad de Ciencias Agropecuarias y Medioambiente, Universidad de La Frontera, Temuco 4780000, Chile

**Keywords:** sperm membrane, cryopreservation, reproduction biotechnology, cold shock, cryoprotectants

## Abstract

Sperm cryopreservation is a fundamental reproductive biotechnology, enabling the long-term storage of genetic material and supporting assisted reproduction programs. Despite its widespread application, the process is associated with significant limitations due to the cryo-induced cellular damage that occurs during freezing and thawing. These injuries primarily affect the plasma membrane, nuclear DNA, and motility, thereby compromising the fertilizing potential of spermatozoa. Furthermore, interspecies variability in terms of cryo-sensitivity poses a major challenge to the development of standardized cryopreservation protocols. Recent advances have focused on mitigating cryodamage through the use of various strategies. The inclusion of antioxidants in cryopreservation media has proven effective in reducing oxidative stress, thereby enhancing cellular protection. Similarly, the addition of lipid-based supplements contributes to membrane stabilization, improving post-thaw sperm viability and functionality. Moreover, the application of omics technologies, such as transcriptomics and proteomics, has facilitated a deeper understanding of molecular damage and protective responses, paving the way for the development of tailored, species-specific protocols. These integrated approaches optimize cryopreservation conditions, maximizing post-thaw survival and the fertilizing capacity of sperm. Enhancing cryopreservation techniques not only improves the outcomes of assisted reproductive technologies, but also plays a crucial role in the conservation of genetically valuable livestock species. In conclusion, the integration of biotechnological and molecular tools holds significant promise for overcoming the current limitations and advancing the efficacy of sperm cryopreservation.

## Simple Summary

Sperm freezing is a fundamental technique in animal reproduction that enables the long-term storage of sperm cells, supporting the improvement of breeding programs and the preservation of valuable genetic traits. However, the freezing and thawing process can cause significant damage to sperm, reducing their ability to fertilize eggs. This damage primarily affects the cell membrane, DNA, and motility, and the extent of the harm can vary depending on the animal species. In this study, we reviewed how freezing impacts sperm from cattle, sheep, horses, and pigs, and explored why some species are more sensitive than others. We also highlighted new strategies scientists are using to minimize this damage, such as incorporating antioxidants and applying advanced tools to better understand molecular responses. Innovative approaches include the use of nanoparticles, exposure to magnetic fields, and a rapid freezing technique known as vitrification. These methods aim to preserve sperm health after thawing, thereby increasing the likelihood of successful fertilization. Enhancing cryopreservation techniques is crucial not only for improving livestock production and animal health but also for conserving endangered species and ensuring global food security.

## 1. Introduction

Sperm cryopreservation is a cornerstone procedure in the advancement of assisted reproductive biotechnologies. It plays a crucial role in both farm animal breeding and the preservation of endangered species, offering a powerful tool for maintaining genetic diversity. This process is inherently complex, involving various protocols, freezing media, and cryoprotective agents [1]. The long-term storage enabled by cryopreservation allows for the transport of sperm across vast distances, facilitates quarantine measures, and even ensures the availability of superior genetic material after the donor’s death, underscoring its significant impact on farm animal production [2].

Despite its advantages, sperm cryopreservation presents challenges, primarily due to heat shock, osmotic imbalance, and intracellular ice crystal formation [3]. Several cryobiological studies have demonstrated that these factors, which compromise sperm survival at low temperatures, often disrupt the integrity of the sperm membrane [4]. The most severe damage is typically seen in the plasma and outer acrosomal membranes during the freeze–thaw cycle, as these structures are most exposed to the cryogenic environment [5,6]. Cold shock-induced damage leads to ultrastructural alterations to the sperm membrane, which is reflected in decreased parameters, such as motility, viability, and membrane integrity, ultimately resulting in a reduction in the fertilization potential of the sperm [7,8].

To mitigate this damage, conventional strategies incorporate chemical additives, including traditional cryoprotectants like glycerol, along with antioxidants, fatty acids, sugars, amino acids, and membrane stabilizers [9]. After cryoprotectant incorporation, the sperm are subjected to controlled cooling, at a rate depending on the species, ultimately reaching subzero temperatures that induce the formation of a viscous cryoprotectant solution during the aqueous phase of the system involving the sperm cell. Subsequently, the cryopreserved sperm are stored at extremely low temperatures, in liquid nitrogen.

The use of frozen sperm in artificial insemination (AI) has become pivotal for improving domestic animal species production potential. However, despite more than 60 years of research, 40–50% of the sperm population does not survive cryopreservation, even when using “optimized” protocols [9,10,11]. Moreover, the extent of this loss varies among species. For instance, in cattle, approximately 60% of the sperm population fails to retain its functionality after thawing [12], while in horses, this proportion may be even higher, with loss rates exceeding 70% [13]. These differences reflect the varying capacity of sperm to adapt to cryopreservation and thawing processes, which differs between individuals within the same species, and even within the same individual within the same species [14]. The reduction in sperm viability negatively affects fertilization rates, resulting in lower conception rates. This loss of viability can have significant economic repercussions, as it requires additional insemination cycles and reduces embryo quality, thereby increasing the costs for farms and veterinary services.

Sperm cryopreservation in domestic animals is a complex procedure that requires the precise regulation of multiple factors to obtain optimal results. Therefore, a thorough understanding of species-specific sperm physiology is essential, as it varies significantly between species and even between individuals in the same species. This review focuses on the membrane damage induced by cryopreservation in key species, such as bovine, ovine, equine, and porcine species, and highlights possible techniques that could be employed to mitigate these detrimental effects.

## 2. Impact on the Sperm Membrane During Cryopreservation

Sperm processing, including cryopreservation, places considerable stress on sperm, primarily affecting sperm membranes [15]. The integrity of the plasma membrane (PM) and acrosomal membrane (AM) are two of the most studied parameters in sperm assessments, due to their crucial roles as cellular boundaries and their role in enabling effective cell–cell interactions, both in terms of morphological and functional integrity [16]. Hence, in regard to the structural evaluation of the spermatozoon, the assessment of the integrity of the PM is emphasized [17].

The sperm membrane is predominantly composed of a lipid bilayer, including phospholipids and cholesterol, as well as various proteins [18,19]. In mammals, the phospholipid composition of the plasma membrane is asymmetric, with phosphatidylcholine and sphingomyelin concentrated in the outer layer, and phosphatidylserine and phosphatidylethanolamine in the inner layer [20]. Glycerophospholipids (GPLs) constitute the most significant lipid fraction of the sperm plasma membrane, with phosphatidylcholine and phosphatidylethanolamine being the most abundant species, and no major qualitative differences observed between species [21,22]. However, significant quantitative differences exist, particularly in regard to the cholesterol-to-phospholipid ratio, the protein-to-phospholipid ratio, the lipid content of the bilayer, and the degree of fatty acid saturation within the GPLs [23]. Understanding the lipid composition and organization of the sperm membrane is essential in order to explain the interspecies variability in terms of its susceptibility to cryopreservation-induced damage. This knowledge not only enhances our basic understanding of sperm biology, but also paves the way for the development of more effective and tailored cryoprotective strategies.

Sperm membrane lipids can exist in a rigid and ordered state (gel type) or in a more flexible and relatively disordered state (fluid type) [21]. Membrane fluidity is essential for the proper functioning of sperm and is influenced by several factors, such as phospholipid and cholesterol concentrations, as well as membrane temperature.

Alterations in membrane composition or dynamics during cooling and freezing processes can compromise sperm function, leading to a reduction in the fertilizing capacity of sperm after thawing. During freezing, the liquid crystalline state transitions to a gel state, wherein fatty acid chains become disordered [24]. This reorganization results in a rigid structure, as fatty acid chains align in a parallel manner. The composition of the plasma membrane not only affects the membrane fluidity and fertilization capability of sperm, but also significantly contributes to the cell’s cryoresistance [25]. Potential membrane damage can lead to a loss of sperm viability. This means that while sperm may survive the freeze–thaw process, their ability to maintain their functionality and fertility may be compromised. Significant changes in sperm lipids occur during cryopreservation, with sperm losing considerable amounts of phosphatidylcholine, phosphatidylethanolamine, and cholesterol post-freezing [26,27]. Furthermore, a decrease in sphingomyelin has been reported following cryopreservation, along with alterations in fatty acids, such as octadecanoate, which decreases, and an increase in polyunsaturated fatty acids (PUFAs), such as docosatetraenoic acid and arachidonate, in various phospholipids [28]. In addition, the freeze/thaw cycle can activate enzymes, such as phospholipase A2 and sphingomyelinase, which generate compounds, such as lysophosphatidylcholine and ceramides, negatively affecting membrane stability and functionality [29]. Membrane fluidity also determines sperm vulnerability during cryopreservation. Therefore, a deep understanding of membrane lipid dynamics enables the identification of key damage sites and potential biochemical targets for the development of more specific and effective cryoprotective agents.

Consequently, lipid–protein interactions, which are essential for proper membrane function, become disrupted [24]. In parallel, certain proteins undergo changes in their localization. For instance, the lectin, SL15, which plays key roles in sperm capacitation, motility, viability, oviductal reservoir formation, and the sperm–oocyte interaction, is reduced in llamas [30]. Similarly, heat shock protein 70 (HSP70), located in the apical region of the buffalo sperm head, plays a crucial role in mammalian fertilization and early embryonic development [31]. Moreover, the alpha 2 subunit of casein kinase 2 (CK2α′), identified in rams, has been associated with DNA damage and the loss of acrosomal integrity [32]. These alterations may lead to an irreversible loss of sperm functionality [33,34].

These alterations are also reflected in the decreased cholesterol content in the sperm plasma membrane (PM). During cryopreservation, temperature-induced phase transitions alter the lipid ordering of the bilayer, disrupting the affinity between cholesterol and membrane phospholipids. As a result, cholesterol becomes more mobile within the bilayer and redistributes toward destabilized regions. Once dislodged, it diffuses into the extracellular milieu.

At the molecular level, this cholesterol efflux is facilitated by membrane transporter proteins, particularly those of the ATP-binding cassette (ABC) family, such as ABCA1 and ABCG1 [35]. These transporters utilize ATP hydrolysis to extract cholesterol from the lipid bilayer and transfer it to extracellular lipid acceptors. The loss of stable cholesterol–phospholipid interactions during cryopreservation increases membrane fluidity and enhances cholesterol mobility, thereby facilitating its recognition and transport by these proteins.

Cholesterol, the predominant lipid in cellular membranes, performs multiple essential functions: it stabilizes membrane structure, reduces permeability, maintains morphological integrity, and mediates cell-to-cell interactions [36]. Additionally, it regulates phase transitions and provides the appropriate physicochemical environment for the proper functioning of integral membrane proteins [37]. Following cryopreservation, cholesterol levels are significantly reduced compared to those in fresh sperm [22], contributing to the structural and functional destabilization of the sperm cell.

Following these modifications to the PM, the dysregulation of lipids occurs, shifting towards the crystalline phase with lateral segregation, which results in oxidative stress [38]. One of the possible mechanisms of sperm cryodamage is the development of oxidative stress, an imbalance between the production of reactive oxygen (ROS) and nitrogen (RNS) species in cells during cryopreservation and the antioxidant defense system. The level of ROS increases, and antioxidant defenses are diminished, due to the centrifugation process that removes seminal plasma before freezing. In addition, sperm cells have very limited cytoplasm, which restricts their intrinsic antioxidant capacity. This combination leads to oxidative damage to crucial biomolecules in reproductive cells, ultimately reducing sperm fertility [39].

**Figure 1:**
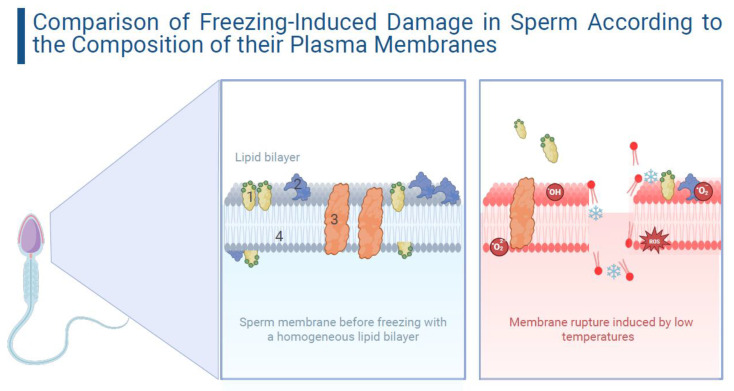
Comparison of the plasma membrane structure of sperm before and after freezing. On the left, a plasma membrane before freezing, with a homogeneous lipid bilayer. On the right, it can be seen how freezing causes structural alterations, such as alteration of membrane fluidity, rupture of the lipid bilayer, and loss of proteins and cholesterol. The elements in the figure are numbered as follows: 1—cholesterol, 2—peripheral protein, 3—integral protein, and 4—phospholipids (including both heads and tails, which together compose the lipid bilayer). Created using BioRender.com.

ROS play a dual role in sperm physiology. At low and controlled levels, ROS trigger redox signaling, necessary for physiological processes that ensure fertilization [40,41]. ROS, such as the superoxide anion (O_2_^−^), hydrogen peroxide (H_2_O_2_), and hydroxyl radical (•OH), act as second messengers in sperm physiology [42]. At physiological levels, they facilitate capacitation, and activate intracellular cyclic adenosine monophosphate (cAMP) levels, which, in turn, induce the activation of protein kinase A (PKA), which, in turn, phosphorylates MEK-like proteins (mitogen-activated protein kinases), threonine–glutamate–tyrosine, and fibrous sheath proteins [43,44]. These signaling cascades lead to final sperm capacitation, making the sperm fully prepared for the acrosome reaction [44,45]. However, at high concentrations, ROS induce oxidative stress, damaging lipids through lipid peroxidation, fragmenting sperm DNA, altering the mitochondrial membrane potential (MMP), triggering apoptosis, and, consequently, leading to infertility [46,47,48,49,50]. When ROS exceed the cell’s antioxidant capacity (mainly in regard to reduced glutathione, superoxide dismutase, catalase, and peroxiredoxins), a chain reaction is initiated that ultimately results in lipid peroxidation (LPO) [51]. During this process, free radicals extract a hydrogen atom from the side chain of a polyunsaturated fatty acid (PUFA), generating a carbon-centered radical [52]. This damage affects nearly 60% of membrane fatty acids, decreasing membrane fluidity, increasing nonspecific ion permeability, and inhibiting the function of membrane receptors and enzymes, perpetuating the damage [47] (Figure 1). These findings highlight that sperm damage is not only structural, but also profoundly functional and biochemical. The loss of key proteins, alterations in lipid dynamics, and oxidative stress, create a hostile environment for the sperm, even if it survives cryopreservation. Understanding these mechanisms enables the design of more precise antioxidant and cryoprotective strategies to preserve sperm fertility.

## 3. Specific Variations in Sperm Responses to Cryopreservation

In the following sections, we analyze how cryopreservation differentially affects sperm depending on the species of interest. Although all sperm cells are susceptible to cryodamage, each species exhibits unique particularities, due to the specific characteristics of their plasma membrane, lipid composition, and susceptibility to oxidative stress (Table 1). These differences determine how damage manifests in regard to sperm functionality and quality, impacting capacitation, the acrosome reaction, and fertilization. Therefore, interspecies variations highlight the need for tailored protocols to optimize cryopreservation and minimize its impact on embryo formation and development. Both ruminant and monogastric species are addressed, considering their distinct reproductive physiology and cryosensitivity.

### 3.1. Cryopreservation-Induced Damage in Ruminant Sperm: Focus on Bovine and Ovine Species

In bovines, cryopreservation represents a significant challenge in regard to preserving sperm functionality. Studies have shown that the PM of bovine sperm contains a lower proportion of cholesterol compared to more cold-resistant species, such as rabbits and humans, specifically the cholesterol/phospholipid molar ratio in bulls is only 0.45, compared to values close to 1.0 in species more tolerant to cooling [53]. This lower cholesterol proportion contributes to greater susceptibility to temperature changes during freezing and thawing.

This lipid composition directly affects protein interactions within the membrane, altering its functionality. During the cryogenic process, a significant portion of surface and membrane proteins is lost or redistributed, affecting their biological function [34]. Key proteins, like P25b and HSP70, which are crucial for fertilization and embryo development, are notably reduced after the freezing process [31,51,54]. The structural rearrangement of the membrane also facilitates the uncontrolled entry of calcium ions, activating the cAMP pathway and associated kinases, which leads to premature capacitation [55]. This early activation compromises the cellular energy balance, depleting the reserves needed to reach and fertilize the oocyte. As a result, a decrease in viability and motility of up to 50%, and damage to the acrosome, ultimately cause a reduction in fertility [56].

Cryopreservation induces an overproduction of ROS, such as O_2_^−^, H_2_O_2_, and OH^−^, which damage critical cellular components, including lipids, proteins, and sperm DNA [1,43]. The ROS generated during the freeze–thaw process cause LPO in the sperm plasma membrane, leading to the formation of toxic byproducts, such as malondialdehyde (MDA), conjugated dienes, and lipid hydroperoxides (including 4-hydroxynonenal [4-HNE] and various 2-alkenals), which exert deleterious effects on membrane integrity [57]. Moreover, these toxic byproducts can inhibit several essential cellular enzymes, such as glyceraldehyde-3-phosphate dehydrogenase (G-3-PDH) and adenosine triphosphatase (ATPase), negatively impacting energy metabolism and potentially compromising sperm motility [58].

These types of damage directly affect the membrane’s fluidity and lipid asymmetry, reduce MMP, and lead to cholesterol redistribution, thereby contributing to acrosomal destabilization and negatively impacting sperm functionality [59]. Furthermore, this leads to the activation of apoptotic pathways through caspases and the increased expression of cell stress proteins like HSP70 [60,61]. These cellular responses contribute to the loss of sperm viability after cryopreservation, negatively impacting reproductive success. For example, in bovines, the use of frozen sperm has been associated with pregnancy rates of 41%, compared to nearly 60% achieved with refrigerated sperm [62]. This lower pregnancy rate with frozen sperm can also be observed in other species (Table 2).

Cryopreservation in sheep presents significant limitations. Artificial insemination (AI) using frozen–thawed ram sperm is not as widely practiced as in other domestic species due to the low and variable fertility rates obtained, as well as the complexity involved in implementing certain technical improvements. Commercial artificial insemination protocols are generally categorized into two main types: those employing chilled sperm (maintained at 15 °C) for vaginal or intracervical insemination, and those utilizing thawed sperm for intrauterine deposition [63]. The latter is a minimally invasive procedure, but remains a surgical intervention that requires veterinary expertise and raises animal welfare concerns, as well as demanding specialized equipment and more manpower compared to cervical insemination [64].

Cryopreservation affects ovine sperm more significantly compared to bovine sperm. After thawing, sperm motility, acrosomal membrane integrity, and the cleavage rate are lower in sheep, with values of 40.3%, 89.0%, and 49.2%, respectively, while in cattle these values are 44.6%, 94.0%, and 62.9%, respectively. This indicates that ovine sperm are more sensitive to freezing-induced damage, negatively impacting their motility, acrosomal membrane integrity, and early embryonic development [65].

Other parameters are also adversely affected: up to 82% of sperm show compromised plasma membrane integrity [66], the mitochondrial function is reduced by 30% after thawing [12], and a slight increase (1.5%) has been observed in the proportion of viable sperm with elevated levels of reactive oxygen species (ROS), such as hydrogen peroxide (H_2_O_2_) and superoxide (O_2_^−^) [67]. Additionally, 14% of frozen–thawed sperm present DNA fragmentation, a value similar to that reported in equine sperm (12%) [68,69].

These findings highlight the need to optimize cryopreservation conditions and insemination techniques in regard to this species. This is even more relevant considering that sheep are seasonal breeders, which limits the availability of fresh sperm to specific periods of the year. In this context, cryopreservation represents a key tool to ensure the availability of high-quality sperm samples year round, allowing for more efficient and flexible reproductive planning.

### 3.2. Cryopreservation-Induced Damage in Ruminant Sperm: Focus on Equine and Porcine Species

The quality of frozen–thawed stallion sperm is lower than that of fresh or refrigerated sperm [70]. Although this phenomenon is observed across all species, what distinguishes horses from other species is that the percentage of viable sperm after thawing is considerably lower, usually ranging from 30% to 50%, compared to other species, such as cattle [71,72,73]. Additionally, the fatty acid composition in the sperm membrane varies according to the reproductive season, being more favorable during spring and early summer. During this period, there is an increase in the levels of PUFAs, which have been associated with higher cell viability and better tolerance to the cryopreservation process [74,75].

During cryopreservation, equine sperm undergoes oxidative stress, membrane damage, DNA fragmentation, a reduction in phospholipase C zeta 1 (PLCZ1) content, and, in some cases, cell death, factors that compromise its post-thaw viability [76,77]. Post-thaw motility ranges from 30% to 60%, and the sperm is frequently rendered unusable due to extremely low motility [13]. Additionally, there is high individual variability: approximately one-third of all stallions produce sperm that is nearly unusable due to low motility and viability after freezing and thawing. As a result, stallions are classified as “good” or “poor” freezers [76,78], with poor freezers showing a high proportion of dead, immotile, or slowly motile sperm (approximately 50%) and a low percentage of progressively motile sperm (<30%) [78]. When sperm exhibit low motility, and semen availability is limited in species wherein individuals have high economic value, such as horses, intracytoplasmic sperm injection (ICSI) is recommended and commonly used to maximize the utilization of stallion sperm [79].

In boar, sensitivity to cryopreservation is particularly pronounced, which has limited its application to less than 1% of artificial inseminations worldwide [80]. This low utilization is due to the severe damage that the sperm suffer during the freezing process, affecting their function, quality, and post-insemination reproductive performance [81,82]. Porcine sperm cells possess a plasma membrane rich in PUFAs and low cholesterol levels, making them highly vulnerable to cold shock and oxidative stress [83,84]. These conditions cause alterations in membrane integrity, the dysregulation of intracellular calcium, acrosomal damage, and changes in lipid distribution [85,86]. At the intracellular level, damage to the perinuclear theca has also been observed, compromising mitochondrial function. This damage alters both the expression and localization of mitofusin-2, a key protein for mitochondrial fusion, as well as the actin network. Furthermore, tyrosine phosphorylation patterns are modified, affecting various fundamental cellular processes [87,88]. At the molecular level, cryopreservation, while useful for preserving cells and tissues, can induce apoptosis (programmed cell death) by triggering the expression of pro-apoptotic proteins like BAX and reducing levels of anti-apoptotic proteins like BCL-2, ultimately compromising cell viability [89]. This process can lead to cell destruction and is a significant consideration in regard to the use of cryopreservation techniques. High levels of ROS and RNS, particularly (O_2_^−^, H_2_O_2_, NO, and peroxynitrite (-ONOO), can negatively affect sperm motility, viability, and physiology by interacting with key cellular components, such as membrane lipids, proteins, and DNA [90,91,92,93]. In this context, H_2_O_2_ has been identified as one of the main ROS responsible for oxidative stress in boar sperm, reducing total sperm motility by 10%, inducing apoptosis in most cells, and causing a 55% decrease in viability, highlighting the high susceptibility of boar sperm to oxidative damage [94].

Alterations in the sperm membrane and ion channels during the freezing and thawing process trigger biochemical and functional changes characteristic of sperm capacitation, even before the sperm cells enter the female reproductive tract. This results in premature capacitation, characterized by early membrane depolarization and asynchronous hyperactivation relative to ovulation [86]. In this regard, it has been shown that piR-121380 regulates ERK2 phosphorylation via its interaction with PTPN7, inducing sperm cryo-capacitation and, ultimately, affecting motility and the fertilizing potential of thawed sperm [95]. Consequently, this entire set of structural, functional, and molecular alterations leads to cumulative damage that compromises sperm viability and fertilizing capacity. A study evaluated the conception rate and litter size in multiparous sows following fixed-time intrauterine insemination, using extended fresh sperm semen and frozen–thawed sperm. Sows inseminated with extended fresh sperm showed a conception rate of 88.9% and an average of 10.8 piglets per litter, whereas those inseminated with frozen–thawed sperm had a conception rate of 75.8% and an average of 9.0 piglets per litter [96].

These results indicate that, although insemination with frozen–thawed sperm may result in slightly lower conception rates and litter sizes compared to extended fresh sperm, it remains a viable option in swine reproduction programs, especially when appropriate insemination techniques and reproductive management are employed.

**Table 2:** Pregnancy rates with fresh and frozen–thawed sperm in farm animals.

Species	Pregnancy Rate with Fresh Sperm (%)	Pregnancy Rate with Frozen–Thawed Sperm (%)	Reference
Cattle	70%	40–50%	[62,97]
Sheep	65–75%	58%	[98,99]
Equine	63%	48.6%	[100]
Porcine	88.9%	75.8%	[96]
Goat	70.4%	59.1%	[101]

## 4. Strategies to Combat Sperm Membrane Damage

In recent years, several studies have proposed innovative approaches to mitigate the damage associated with bovine sperm cryopreservation, particularly regarding sperm membrane integrity and post-thaw fertility. Recent research has evaluated the addition of the granulocyte-macrophage colony-stimulating factor (GM-CSF). This pleiotropic cytokine is naturally present in the sperm of mammals, including livestock sperm [102,103,104]. Several studies have shown that GM-CSF can positively influence the resistance of ram sperm to environmental heat stress [102], as well as improve sperm motility in bovines and in men with oligoasthenoteratozoospermia [103,105]. Recently, it has been demonstrated that the incubation of frozen–thawed bovine sperm with GM-CSF may represent a promising biotechnological tool to enhance reproductive efficiency. This approach has been shown to significantly improve key parameters such as motility, capacitation, and the fertilization rate, in addition to promoting early embryonic development in cattle [106].

The substitution of egg yolk with a cholesterol–cyclodextrin complex represents a highly effective option, especially when the cyclodextrins are preloaded with cholesterol. These agents promote the insertion of cholesterol into cell membranes, due to the presence of a hydrophobic inner core and their high affinity for sterols [107]. Various studies have shown improved cryosurvival rates of sperm from bulls [108,109]. The preservation of PM integrity and in vitro fertilizing ability has also been observed, further supporting its potential as a substitute for egg yolk in cryopreservation protocols [110]. This alternative not only preserves sperm quality, but also represents a safer option from a biosecurity perspective, reducing the risk of the transmission of zoonotic diseases associated with the use of egg yolk.

The combined use of cyclodextrins with natural flavonoids, such as kaempferol (3,4′,5,7-tetrahydroxyflavone) or epicatechin (EPC), constitutes an innovative strategy to mitigate cryopreservation-induced damage in bovine sperm. Cyclodextrins provide structural stability to the PM by modulating its lipid content, while flavonoids act as potent antioxidants capable of neutralizing (ROS), thus reducing oxidative stress that compromises sperm viability. Recent studies have shown that the addition of kaempferol and epicatechin to freezing extenders significantly improves membrane integrity, motility, viability, and sperm morphology after thawing [111,112]. The ability of these compounds to preserve mitochondrial function has also been supported in regard to other species, such as boars, wherein their supplementation in sperm extenders helped maintain key biofunctional parameters during storage [113]. For future research and practical applications in artificial insemination programs, the implementation of these innovative advances in a complementary manner is recommended, aiming to enhance reproductive efficiency, while ensuring the safety and effectiveness of bovine sperm cryopreservation.

To help mitigate the damage caused by cryopreservation in ram sperm, some recent studies have suggested adjusting the sperm concentration and selecting appropriate commercial extenders. According to a study by Paucar Quito [114], lower sperm concentrations (200 and 400 × 10⁶/mL) achieved pregnancy rates of 57.5% and 54.4%, respectively, compared to a concentration of 800 × 10⁶/mL (45.5%). In the same study, three commercial extenders, Ovixcell, Andromed, and Triladyl, were evaluated. It was concluded that Triladyl was the most effective in preserving the functional characteristics of ovine sperm, protecting the plasma membrane and offering greater resistance to the harmful effects of cryopreservation. The ProAKAP4 protein, along with its precursor AKAP4, is one of the most abundant in the principal piece of the sperm flagellum and plays a fundamental role in motility. ProAKAP4 has already been validated as a molecular marker of sperm quality in several species, including mice, boars, and humans, due to its strong correlation with functional parameters, such as motility and fertility [115,116,117,118,119]. In rams, sperm samples from fresh, cooled, and cryopreserved conditions were evaluated, and the results showed that cryopreservation significantly reduced sperm quality, with a marked decrease in the ProAKAP4 concentration [120]. Based on the reviewed evidence, these strategies can be useful for improving post-thaw ovine sperm quality and viability, thereby optimizing outcomes of artificial insemination and fertilization programs. Incorporating ProAKAP4 assessments into routine ovine sperm quality analysis is recommended, especially for cold storage and cryopreservation protocols. This tool, combined with antioxidant strategies and the appropriate choice of extenders and sperm concentration, could significantly increase success rates in regard to ovine artificial insemination programs.

**Figure 2:**
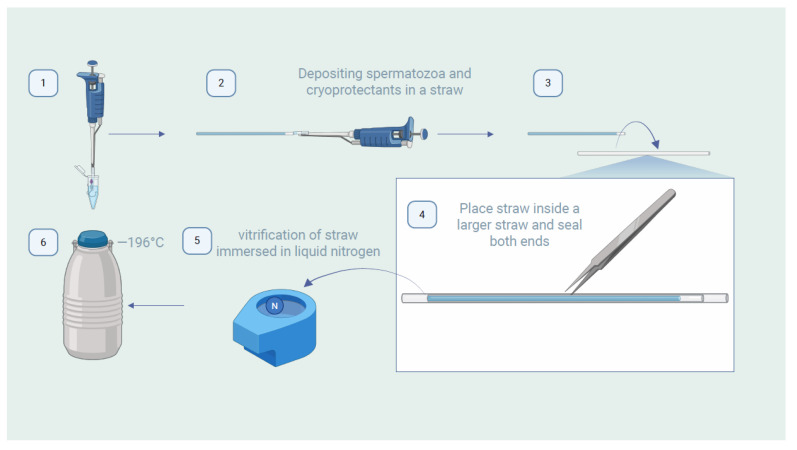
Sperm vitrification process step by step: (1) preparation of the cryoprotectant solution, (2) deposit the sperm sample in the straw horizontally, (3) place this straw inside a larger straw and seal the edges to prevent contamination, (4) use forceps in the center to handle the straw, (5) deposit the straw in a container with liquid nitrogen for rapid freezing, and (6) store the straw at −196 °C for long-term preservation. This procedure ensures efficient preservation of the sperm. Created using BioRender.com.

To improve the quality of frozen stallion sperm, various approaches have been explored, such as the use of antioxidants like hydroxytyrosol (HT) and resveratrol (RSV), which help reduce damage caused by oxidative stress, enhance plasma and acrosomal membrane integrity, and improve post-thaw motility [121,122]. However, individual stallion variation significantly influences sperm survival during cryopreservation, highlighting the importance of tailoring freezing protocols to each individual to optimize outcomes [123]. A key aspect is the sperm concentration, which directly impacts sperm quality after preservation. In this context, when sperm is highly concentrated, it must be adequately diluted to reach the optimal concentration before use [71]. The recommended minimum concentration of progressively motile sperm (PMS) for artificial insemination (AI) with frozen sperm is at least 250 × 10⁶ sperm per dose. An innovative alternative is the use of vitrification, a process that produces a glass-like solidification of living cells that completely prevents the formation of ice crystals during cooling. Equally important, the vitrification process completely prevents the formation of ice crystals in cryopreserved cells during heating, in order to recover the cells for biological applications. It is a process that enables the direct transition of an aqueous solution from a liquid to a glassy state, thereby avoiding ice crystal formation [124,125]. This rapid technique minimizes cold damage and has shown promising results in humans, dogs, and cats [1]. In humans, sperm vitrification has been studied and optimized for over a decade, showing greater cryoresistance compared to other species. In equines, vitrification represents an ideal alternative to conventional freezing. Recent studies have shown that vitrified sperm without permeable cryoprotectants, using 0.25 mL straws containing 100 μL of sperm at a concentration of 100 × 10⁶ sperm/mL and an extender with 100 mM trehalose, exhibit better post-thaw parameters than sperm conventionally frozen with glycerol [126]. Another study used the same straw technique (0.25 mL) with protective outer covers, ensuring sterile conditions during the process and reinforcing its practical applicability. In horses, significantly higher progressive motility was observed in vitrified sperm (48.2 ± 2.3%) compared to conventionally frozen sperm (37.3 ± 2.2%). Moreover, in donkeys, while pregnancy rates were similar between vitrified (22%) and frozen sperm (10%), uterine post-insemination inflammatory responses resolved more quickly when vitrified sperm was used [127]. These findings suggest that vitrification not only improves post-thaw sperm quality, but may also reduce adverse effects associated with artificial insemination in equines (Figure 2).

The cryopreservation of porcine sperm continues to face significant challenges, due to the sensitivity of the sperm to the physical and chemical changes induced by the freezing and thawing process. In this context, a direct correlation has been identified between post-thaw sperm quality and sensitivity to oxidative stress induced by hydrogen peroxide (H_2_O_2_) prior to freezing. This finding suggests that evaluating the sperm response to H_2_O_2_ could serve as a predictor of cryotolerance in porcine sperm [128]. To mitigate these negative effects, various strategies have been developed, with promising results. For example, the inclusion of glutamine in the sperm extender during storage at 17 °C prior to freezing has been shown to significantly improve post-thaw motility, viability, and the mitochondrial activity of sperm [129]. Likewise, the protective effects of betaine during liquid storage and sperm transport have been evaluated. Concentrations of 0.5 mg/mL significantly preserved sperm motility during storage at 17 °C for 3 to 5 days, while a concentration of 2.5 mg/mL improved sperm progressive motility during road transport. Furthermore, 0.4 mg/mL of betaine was found to enhance the mitochondrial activity, antioxidant capacity, and reduce lipid peroxidation damage [130]. Another innovative strategy involves exposing the freezing extender to magnetic fields of up to 6000 Gauss (G), which resulted in a significant reduction in sperm membrane damage and increased post-thaw motility [131]. In line with antioxidant-based approaches, the use of functionalized nanoparticles has also been explored. For example, epigallocatechin gallate (EGCG) was incorporated into a polydopamine (PDA). Thanks to their ability to disassemble into ultrasmall, bio-targeted particles (<10 nm), this rationally designed nanoprotector ensures prolonged antioxidant activity and specificity in regulating protein phosphorylation within boar semen preservation systems at 4 °C. Beyond enhancing antioxidant protection, these nanoparticles also modulate intracellular signaling pathways, such as the D2DR/cAMP/PKA cascade, thereby extending sperm viability from 3 to up to 10 days at 4 °C, a significant advancement compared to conventional antioxidants [132]. By simultaneously enhancing the antioxidant defense and modulating key intracellular signaling pathways, this approach represents a paradigm shift in the field of sperm cryobiology and liquid storage, opening new avenues for precision sperm preservation strategies across domestic species. From a molecular perspective, proteins such as α-amylase and epididymal sperm-binding protein 1 have been identified as potential biomarkers of boar sperm freezing resistance. Supplementation with α-amylase in regard to the cryoprotective extender has been shown to significantly enhance motility and sperm quality after thawing [129]. Finally, sperm storage at 4 °C using specialized extenders, such as AndroStar Premium, has been explored as an alternative to conventional storage at 16–18 °C. This method has been proven to be effective in maintaining DNA integrity and in vitro fertilizing capacity, opening up new possibilities for enhancing the functional longevity of stored sperm [133].

## 5. Future Projections

In the coming years, research is expected to increasingly focus on deciphering the molecular signatures that confer cryotolerance, particularly those related to sperm membrane stability. Proteomics will play a pivotal role in identifying membrane-associated proteins that either protect or compromise sperm viability during freezing and thawing processes. A promising avenue lies in modulating the lipid composition and in the targeted supplementation of cryoprotectants that specifically interact with membrane lipids and proteins to preserve their structure and function. Furthermore, the integration of proteomics with lipidomics and transcriptomics may lead to the development of customized extenders or nanoparticle-based delivery systems that enhance membrane resilience. These advancements hold the potential to revolutionize cryopreservation strategies, making them more species-specific and efficient, while also paving the way for precision breeding and the conservation of valuable genetic traits.

## 6. Conclusions

Sperm cryopreservation, while essential for reproductive biotechnologies, continues to face significant limitations, due to cellular damage induced by the freezing and thawing process. These effects vary across species, affecting everything from membrane integrity to fertilization capacity. However, recent advances in the use of antioxidants, lipid-based supplements, and omics technologies present new opportunities to optimize protocols and enhance post-thaw fertility. A deep understanding of the mechanisms of damage and protective strategies will enable the development of more effective and species-specific methods, contributing to the success of assisted reproduction programs and the genetic conservation of animals of zootechnical interest.

## Figures and Tables

**Table 1:** Interspecies comparison of sperm membrane properties and cryopreservation-induced damage in domestic species.

Species	Cholesterol/Phospholipid	Membrane Fluidity	Susceptibility to Cold Shock	Post-Thaw Motility (%)	Main Cryopreservation Damage
Bovine	+++	+	+	>50–70%	Minimal functional changes
Ovine	++	++	++	40–60%	Membrane fragmentation, loss of acrosome
Stallion	++	++	++	30–60%	Oxidative stress, membrane and DNA damage
Boar	+	+++	+++	<30%	Cold shock, apoptosis, mitochondrial damage

The data in this table were extracted and summarized from the main text for clarity and comparative purposes. The relative levels of cholesterol/phospholipid ratio, membrane fluidity, and susceptibility to cold shock are indicated using the following scale: +++ = high, ++ = intermediate, + = low.

## Data Availability

No new data were created or analyzed in this study.
